# Purification and biochemical characterization of a novel thermostable serine alkaline protease from *Aeribacillus pallidus* C10: a potential additive for detergents

**DOI:** 10.1080/14756366.2016.1261131

**Published:** 2017-01-18

**Authors:** Vildan Yildirim, Mustafa Ozkan Baltaci, Ilknur Ozgencli, Melda Sisecioglu, Ahmet Adiguzel, Gulsah Adiguzel

**Affiliations:** a Department of Molecular Biology and Genetics, Faculty of Science, Ataturk University, Erzurum, Turkey;; b Department of Chemistry, Faculty of Science, Ataturk University, Erzurum, Turkey;; c Department of Food Hygiene and Technology, Faculty of Veterinary, Ataturk University, Erzurum, Turkey

**Keywords:** Thermotolerant, *Aeribacillus pallidus*, alkaline serine protease, biochemical characterization, purification

## Abstract

An extracellular thermostable alkaline serine protease enzyme from *Aeribacillus pallidus* C10 (GenBank No: KC333049), was purified 4.85 and 17. 32-fold with a yield of 26.9 and 19.56%, respectively, through DE52 anion exchange and Probond affinity chromatography. The molecular mass of the enzyme was determined through sodium dodecyl sulfate-polyacrylamide gel electrophoresis (SDS-PAGE), with approximately 38.35 kDa. The enzyme exhibited optimum activity at pH 9 and at temperature 60 °C. It was determined that the enzyme had remained stable at the range of pH 7.0–10.0, and that it had preserved more than 80% of its activity at a broad temperature range (20–80 °C). The enzyme activity was found to retain more than 70% and 55% in the presence of organic solvents and commercial detergents, respectively. In addition, it was observed that the enzyme activity had increased in the presence of 5% SDS. *K*_M_ and *V*_max_ values were calculated as 0.197 mg/mL and 7.29 μmol.mL^−^^1^.min^−^^1^, respectively.

## Graphical abstract

**Figure:**
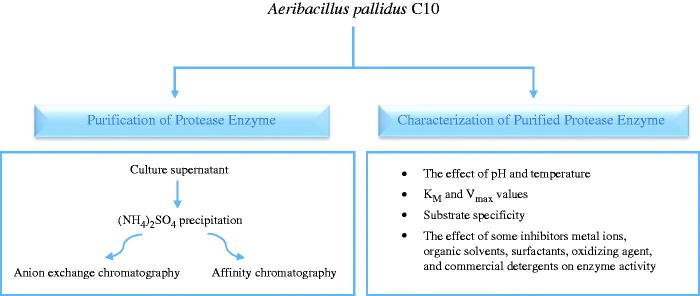

## Introduction

The largest class of commercial enzymes on which industrial applications are done comprise proteolytic enzymes. Proteases or peptidases form the hydrolytic enzyme group which hydrolyzes the peptide bonds within the structure of proteins and catalyzes the peptide synthesis in the presence of an organic solvent^1^^,^ 2. They are classified as acidic, alkaline and neutral proteases according to the pH ranges they are active at; and as serine, aspartic, cysteine and metalloproteases according to the functional group found in its active area^3^^,^ 4. Sixty to sixty-five percent of the global enzyme market, the largest part of which consists of alkaline proteases, is comprised of proteases^5^. Alkaline proteases are commonly used in a number of fields, such as leather, textile, waste management industries as well as food and pharmaceutical industries, notably the detergent industry^2^^,^ 6 ^,^ 7. In the presence of oxidizing agents and anionic surfactants, which are the additives used commonly in the modern detergent formulation, alkaline proteases that are both stable and active in the solutions with high pH, as well, are of importance in the detergent industry in particular^8^. For this reason, in the presence of high specificity, thermostability, alkaline pH and organic solvents, the industrial demand for quite active preparates of proteolytic enyzmes with high stability urges those involved in this field to investigate alternative protease resources.

Scientists are quite intensively interested in alkaline proteases that are produced by microorganisms and are quite important in terms of biotechnology^9^. The reason for this is that the enyzmes originating from microorganisms have high-catalytic activities, that they do not cause any undesired by-product, that their growth conditions can be easily optimized, that they are quite inexpensive, and that they can be produced in quite large amounts with high purity and at a single production process^10^^,^ 11. Apart from the fact that alkaline proteases are produced by a large microorganism group in which fungi and bacteria are also included, a great majority of commercial alkaline proteases in particular are obtained from the bacteria of *Bacillus* sp. species^12^^,^ 13. Thermophilic microorganisms are quite important in terms of industry, since they are capable of adapting to extreme conditions, such as high temperature, pressure, pH and salt concentration^14^. *Aeribacillus pallidus*, a thermophilic bacterium, belongs to the class of *Bacilli* species, and its optimum growth temperature is 55–60 °C^15^. There are many studies in the literature in which the characterization of *A. pallidus,* a type species of this new genus, is performed through conventional and molecular methods^16–20^. Cihan et al.^18^ identified the *Aeribacillus* strain and stated that it had no protease activity, whereas in our study, we determined that the strain we used had a high-protease activity.

No enzymatic research regarding this new species has been found in the literature, and for the first time with our study, a research into the purification, characterization and biotechnological applicability of the protease enzyme from *A. pallidus* C10 strain was performed.

## Materials and methods

### Identification of thermophilic bacteria

The isolation and identification of the test strain used in this study was reported elsewhere^20^.

### 16S rRNA gene sequence analysis

**Figure 1.**
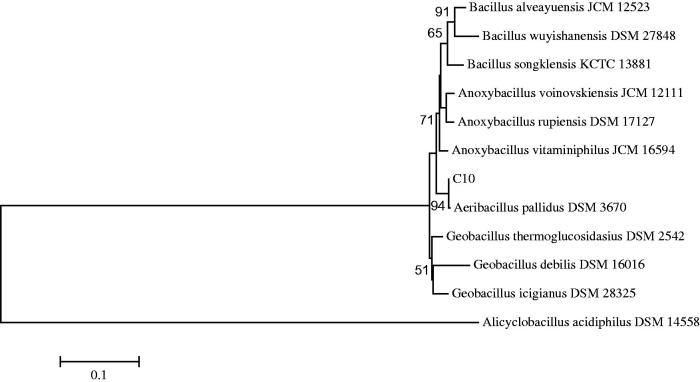
Neighbor-joining phylogenetic tree on the basis of 16S rRNA gene sequence data of the thermophilic bacteria. *Alicyclobacillus acidiphilus* DSM 14558 was used as out-group. Bootstrap values based on 1000 replications are listed as percentages at branching points. Only bootstrap values >50% are shown at nodes. The scale bar represented 1% divergence.

16S rRNA, which is the evolutionary gene of the prokaryotic ribosome, from the genomic DNA of the test isolate, was amplified by PCR with the forward primer UNI16S-L: (5′-ATTCTAGAGTTTGATCATGGCTCA-3′) and the reverse primer UNI16S-R: (5′-ATGGTACCGTGTGACGGGCGG TGTGTA-3′). The amplified fragment was cloned to a vector system (pGEM-T, Promega, the UK); and then, the clone was sequenced (Macrogen, Amsterdam, the Netherlands). The result of 16S rRNA gene sequencing was analyzed using the GenBank and EzTaxon (http://blast.ncbi.nlm.nih.gov/blast.cgi and http://www.eztaxon.org) server. Considering the results of the study, a phylogenetic tree was formed via the Neighbor Joining Method (Figure 1) using the MEGA 4.0 software package (Phoenix, AZ)^21^.

### Enzyme and protein assay

The protease activity was determined through the modification of the method defined by Takami et al.^22^. By adding 2.5 mL from 0.65% of casein solution prepared within 100 mM Tris-HCl buffer (pH 8.5) onto 0.5 mL enzyme solution, the reaction solution was kept at 37 °C for incubation for 10 min. A blank was performed in the same procedure; but this time, 0.5 ml of 100 mM Tris-HCl buffer (pH 8.5) was used instead of the purified enzyme. At the end of the incubation, the reaction was stopped by adding 2.5 mL from 110 mM trichloroacetic acid (TCA). After this mixture was kept at 37 °C in water bath for 30 min, it was centrifuged at 10,000 × *g* for 10 min. By adding 2.5 mL from 0.5 M Na_2_CO_3_ solution and 0.5 mL from 0.5 M Folin-Ciocalteu reagent into 1 mL of the supernatant, the mixture was kept at 37 °C for 30 min after the absorbance was measured at 660 nm. One unit of protease activity was defined as the amount of enzyme required to liberate l μg/min-tyrosine at 37 °C and at pH 8.5. The protein concentration was determined according to the Bradford method, in which bovine serum albumin (BSA) was used as the standard^23^.

### Protease enzyme purification

#### Ammonium sulfate precipitation and dialysis

The cell isolates of *A. pallidus* C10 were centrifuged at 10,000 × *g* for 10 min, and the culture supernatant containing extracellular protease was subjected to ammonium sulfate precipitation. The mixture precipitated through the ammonium sulfate at the range of 0–90% was centrifuged at 5000 × *g* for 15 min. The precipitate obtained was dissolved through 100 mM Tris-HCl (pH = 8.5) buffer solution at minimum volume and was dialyzed against the same buffer.

#### Ion exchange chromatography

The dialyzate was loaded into DEAE-cellulose (DE52, Whatman) column balanced with 50 mM Tris-HCl buffer (pH 8.5), and bound proteins were eluted with a linear gradient of 50–500 mM NaCl in the same buffer. Fractions of 5 mL were collected and analyzed for enzyme activity and protein concentration. Fractions showing maximum activity were pooled and stored at 4 °C for further analysis.

#### Affinity chromatography

A second purification method was performed on the protease enzyme through Probond Nickel Chelating Resin (Invitrogen) affinity chromatography. After ammonium sulfate precipitation had been performed, the dialyzed sample was connected to Probond affinity column equalized with a natural-binding buffer (0.5 M NaCl and 50 mM NaH_2_PO_4_, pH 8.0). After the removal of the proteins which did not contain histidine amino acid by using a wash buffer (0.5 M NaCl, 20 mM imidazole, and 50 mM NaH_2_PO_4_, pH 8.0), the protease enzyme was eluated from the column by using an elution buffer (0.5 M NaCl, 250 mM imidazole, and 50 mM NaH_2_PO_4_, pH 8.0). Enzyme and protein assays were performed on the collected fractions.

### SDS-polyacrylamide gel electrophoresis and zymography

For the control of the purity and determination of molecular weight of the enzyme, sodium dodecyl sulfate-polyacrylamide gel electrophoresis (SDS-PAGE) was performed according to the Laemmli method^24^. The protein bands were determined through the silver-staining process. For the zymogram analysis of alkaline protease enzyme, SDS-PAGE was performed according to a method modified by Garcia-Carreno et al.^25^. After the electrophoresis, the gel was kept in 2.5% TritonX-100 solution, which was prepared with 100 mM pH 8.5 Tris-HCl, in order to remove the SDS in shaker at 37 °C with 150 rpm for 30 min. Afterwards, the gel was washed up three times with 100 mM Tris-HCl (pH 8.5) buffer, and then TritonX-100 was removed. Finally, the gel was incubated in 0.65% casein solution prepared within 100 mM Tris-HCl (pH 8.5) buffer, in a shaker operating at 37 °C and 150 rpm speed. After incubation, silver-staining process was performed.

### The effect of pH on protease enzyme activity and stability

For the purpose of determining the optimum pH of the enzyme, the protease activity was performed by using sodium phosphate (pH 6.0–8.0), Tris-HCl (pH 8.0–9.0) and glycine-NaOH (pH 9.0–11.0) buffer at 0.1 M different pH values containing 0.65% casein. The enzyme was incubated in various buffer solutions (pH 6–11) at room temperature for 2 h for pH stability study, and the residual activity was determined in standard experimental conditions.

### The effect of temperature on protease enzyme activity and stability

The effect of temperature on the enzyme activity was determined by measuring the enzyme activity at pH 8.5 for 10 minutes at different temperatures, from 20 °C up to 80 °C. To determine the temperature stability of the enzyme; the activity was measured by leaving the enzyme solution for incubation at different temperatures (20–80 °C) for 15, 30 and 60-min periods. After the amount of activity prior to the incubation at different temperatures had been accepted as 100, the values obtained in the wake of the incubation were expressed as the residual activity percentage (%).

### Enzyme kinetics

In order to determine *K*_M_ and *V*_max_ values of the protease enzyme purified from *A. pallidus* C10, the activities of the casein substrate at the range of 0.02–0.6% at different concentrations were measured. With the obtained values, the Lineweaver–Burk graphic was drawn, and by making use of this graphic, the kinetic parameters, such as *K*_M_ and *V*_max_, were calculated^24^^,^ 26.

### Substrate specificity

The substrate specificity of C10 protease enzyme was determined by using casein, azocasein, hemoglobin, bovine serum albumin (BSA) and gelatin substrates. 0.65%-solutions pertaining to the substrates were prepared in a 100 mM Tris-HCl (pH 8.5) buffer. Later on, 0.5 mL of enzyme and 2.5 mL of substrate solution were mixed, and the activity was measured. The activity of the enzyme along with the substrate through which it showed the highest activity was accepted as 100%, and the relative enzyme activities were calculated.

### The effect of some inhibitors and metal ions on C10 protease activity

The effect of enzyme inhibitors on the protease activity was determined by using phenylmethylsulfonyl fluoride (PMSF) (2 and 5 mM), ethylenediaminetetraacetic acid (EDTA) (2 and 5 mM), β-mercaptoethanol (1 and 5%), and ditio-bis-nitrobenzoic acid (DTNB) (2 and 5 mM). The purified enzyme was incubated in the presence of protease inhibitors at 37 °C for 30 min, and the amount of the activity of the enzyme solution containing no inhibitor was accepted as 100%, and the residual enzyme activity was calculated.

The effect of the metal ions on the protease enzyme activity was decided on by measuring the enzyme activity in the presence of 1, 5 and 10 mM concentrations of various metal ions (Co^2+^, Mg^2+^, Fe^2+^, Mn^2+^, Ca^2+^, Ni^2+^, Zn^2+^, K^+^, Ag^+^). The activity amount of the enzyme solution containing no metal ion was accepted as 100%, and the activity of the samples containing any metal ion was calculated as the residual activity.

### The effect of some organic solvents on protease stability

In order to examine the change of the protease enzyme in activity, which had been purified from *A. pallidus* C10, in the presence of organic solvents; the enzyme was mixed with organic solvents, such as methanol, ethanol, acetone, DMSO, butanol, chloroform, isopropanol, at 15%, 25%, and 50% concentrations, after which it was incubated for 1 and 24 h. The activity amount of the enzyme solution containing no organic solvent was accepted as 100%, and the activity of the samples containing any organic solvent was calculated as the residual activity.

### The effect of surfactants, oxidizing agent and commercial detergents on the stability of alkaline protease

The effects of some of the surfactants (SDS, Triton X-100, Tween-20, Tween-80) and the oxidizing agent (H_2_O_2_) on the protease activity were determined by subjecting the enzyme along with the surfactants and oxidizing agents to pre-incubation at different concentrations (1 and 5%) at 37 °C for 30 min. The residual activity after incubation was calculated, and the enzyme activity with no additive at all was taken as 100%.

The compliance of the protease purified by using Bingo (Hayat Chemistry, Turkey), Alo (Procter&Gamble, Turkey), Rinso (Unilever, Turkey), Persil (Henkel, Turkey), Ariel (Procter&Gamble, Turkey) and OMO (Unilever, Turkey) with commercial solid laundry detergents was examined. The solutions of the detergents at 1%-concentrations were prepared in tap water. The diluted detergents were heated up at 100 °C for an hour prior to the addition of the enzyme, and the endogenous proteases found within these detergents were inactivated. The enyzme solution along with the detergent solutions prepared was left for incubation at 37 °C for an hour, and the residual activity was calculated. The sample, as a control, in which there was no detergent solution was left for incubation under the same conditions, and the activity of the control was accepted as 100%.

### Statistical analysis

All the experiments were repeated three times, and as for the evaluation of the data, The GraphPad Prism-version 5.00 (Graphpad Software, San Diego, CA) statistical program was used. The mean ± standard error (SEM) (*n* = 3) values seen in all the results were analyzed.

## Results and discussion

### Characterization of the thermophilic bacteria

Some morphological, physiological and biochemical tests were applied to the test strain (C10), which was isolated from Sirnak–Belkisana hot spring in Turkey. According to the results, the isolate was moving rods which formed aerobic, thermophilic, Gram positive, catalase, oxidase, endospore positive. The optimum pH and temperature for the test strain was detected as 9.0 and 55 °C, respectively. This met the criteria of thermophilic bacteria, which reproduced at temperatures above 50 °C^21^. The thermophilic bacteria were able to reproduce at a range of salt concentration of 2–6%.

As a result of 16S rRNA sequence analysis, the test isolate was determined to contain about 1427 nucleotides (nt). Later, in consequence of the blast study carried out by using the database in the Genbank and EzTaxon, the thermophilic bacteria were detected to be the strain belonging to the genus of *Aeribacillus* at a powerful rate of >99% (GenBank No: KC333049).

### Purification of *A. pallidus* C10 protease

The protease enzyme from *A. pallidus* C10, after the ammonium sulfate precipitation (90% w/v), was purified 4.85 and 17.32-fold at 26.9 and 19.56% efficiency, respectively, through the use of DE52 anion-exchange chromatography and Probond affinity chromatography. The purification profile of the protease enzyme is summarized in Table 1. In the purification process performed through two different chromatographic methods in this study, the purification coefficient was observed to have increased when Probond affinity chromatography was used in comparison to the anion-exchange chromatography. It was determined that when these purification results were compared with those in the literature, similar results^27^^,^ 28 were seen and that higher efficiency^29^^,^ 30 and fold purification^27^^,^ 31 were obtained when compared with some other studies. It is seen in the literature that purification processes for proteases have been performed through several different methods, such as ammonium sulfate precipitation, ultrafiltration, DEAE-Sepharose fast flow/Mono Q-Sepharose ion exchange chromatography, Sephadex gel filtration and Sepharose-bacitracin affinity chromatography, or different combinations of these. Nonetheless, although the Nickel-affinity **(**Probond) column which binds histidine amino acids to the nickel ion found within its structure is used to purify rather the recombinant proteins, it can also be used for the natural proteins that have an affinity on their own to divalent cations^32^^,^ 33. This method was used by starting from the fact that purification could be performed through this affinity column in serine proteases that keep histidine amino acid in their catalytic region^34^.

**Table 1.** Summary of the alkaline protease from *A. pallidus* C10 purification procedure.

Purification methods	Purification steps	Total activity(U**)**	Total protein(mg)	Specific activity(U/mg)	Yield (%)	Purificationfold
DE52 anion exchange chromatography	Culture supernatant	2148	42960	0.056	100	1
	(NH_4_)_2_SO_4_ precipitation and dialysis	283.6	1610	0.176	13.02	3.14
	Anion exchange chromatography	76.3	280	0.272	26.9	4.85
Probond affinity chromatography	Culture supernatant	2473.8	40380	0.061	100	1
	(NH_4_)_2_SO_4_ precipitation and dialysis	332.5	4088	0.081	13.44	1.33
	Affinity chromatography	65.04	61.05	1.057	19.56	17.32

**Figure 2.**
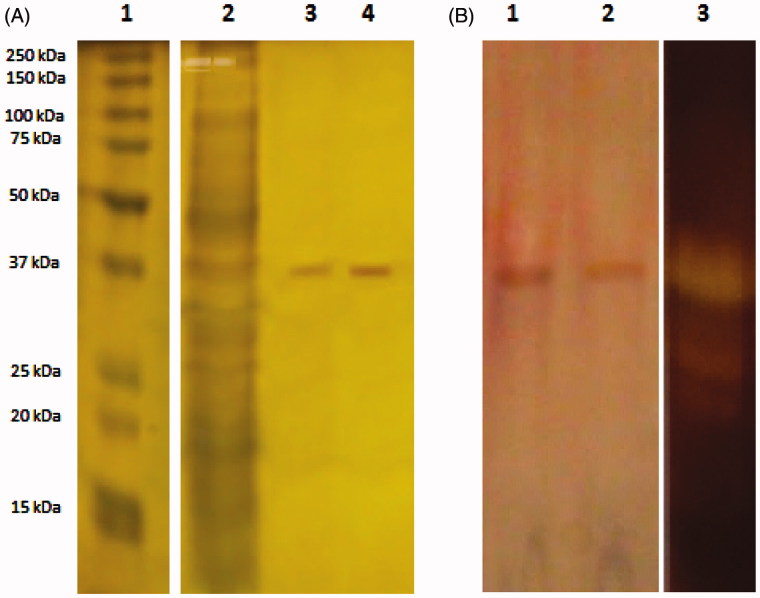
SDS-PAGE of the purified protease from *A. pallidus* C10. (A) Lane 1: Standard protein molecular mass markers, lane 2: (NH_4_)_2_SO_4_ precipitated proteins, lane 3–4: Purified protease from DE52 anion exchange chromatography, (B) Lane 1–2: Purified enzyme from Probond affinity chromatography, lane 3: Zymography of the purified enzyme.

The molecular weight of the purified protease enzyme was estimated to be approximately 38.35 kDa by SDS-PAGE (Figure 2(A,B)). In general, apart form their exceptions; the molecular masses of microbial alkaline proteases vary between 15–36 kDa^8^. In previously-conducted studies, alkaline proteases with molecular weights similar to *A. pallidus* C10, such as *B. pumilis* CBS (34 kDa)^35^, *P. fluorescens* (36 kDa)^36^ *, B. pumilus* MCAS8 (36 kDa)^37^ *, B. lehensis* (39 kDa)^38^; and alkaline proteases with different molecular weights such as *A. nidulans* HA-10 (42 kDa)^39^, *B. cereus* 1173900 *(*66 kDa)^7^ *, B. mojavensis* A21 (20 kDa)^8^ *, B. subtilis DM-04* (16.9 kDa)^27^ were reported. The purity and activity of the enzyme was also evaluated using zymogram activity staining. A clear zone of hydrolysis was observed in the gel indicating the presence of proteolytic activity (Figure 2(B)).

### The effect of pH on alkaline protease activity and stability

**Figure 3.**
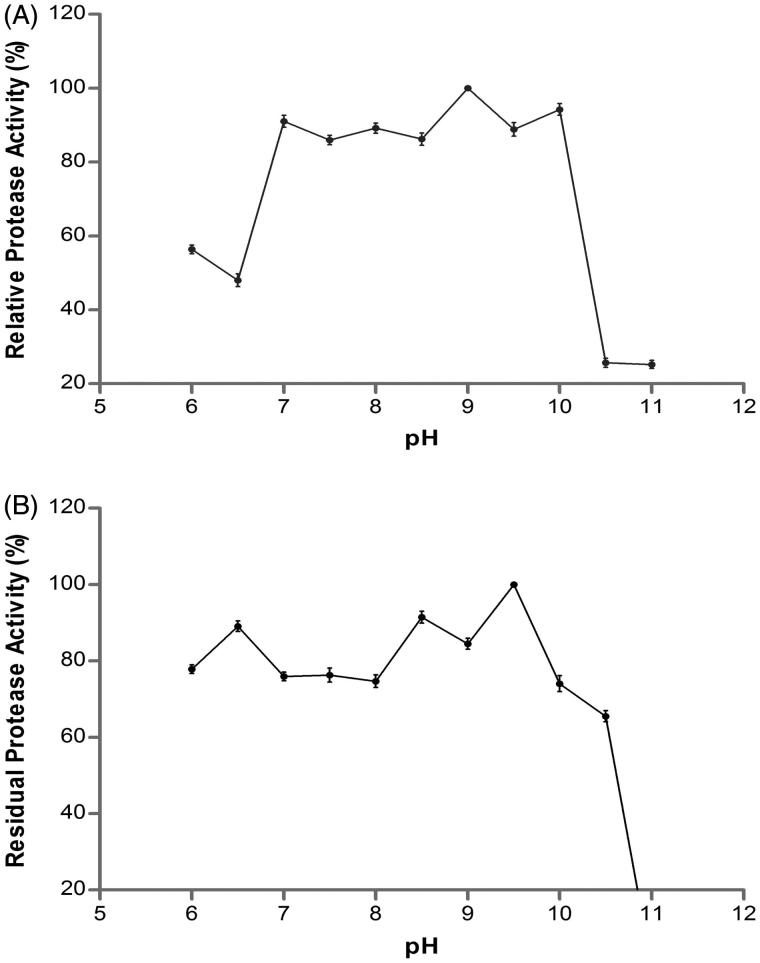
Effect of pH on activity (A) and stability (B) of the purified protease from *A. pallidus* C10.

The optimum pH of the protease enzyme purified from *A. pallidus* C10 with high activity at the range of pH 7–10 was determined as 9.0 (Figure 3(A)). The fact that the enzyme preserves more than 85% of its activity at the range of alkaline pH (7.0–10.0) indicates to the fact that the enzyme in question is an alkaline protease. These findings are in accordance with the proteases, the optimum pH of which was at the range of 7.0–10.0 in the previously reported studies^37^^,^ 40 ^,^ 41.

When the pH stability profile for the protease enzyme purified from *A. pallidus* C10 was examined; it was determined that the enzyme had preserved its activity by more than 70% at the range of pH 6.0–10.5 after the 2-h-incubation at room temperature. The pH stability results showed that the protease was 100% stable at pH 9.5 and that it lost its activity at the rate of 11, 9, 16 and 30% at pH 6.5, 8.5, 9.0, and 10.0, respectively (Figure 3(B)). Some alkaline proteases with similar pH stability ranges have also been reported, such as *B. Pumilus* CBS^35^, *B. amyloliquefaciens* SP1^42^, *Bacillus* strain HUTBS71^43^, and *B. licheniformis* P003^44^. In detergent formulation, when the significance of proteases at high-pH values that are particularly stable at 8.5–10.0 pH range is taken into consideration^12^^,^ 45 ^,^ 46, the fact that *A. pallidus* C10 protease enzyme has an activity and pH stability under high-alkaline conditions indicates to its potential applicability for the detergent industry.

### The effect of temperature on alkaline protease activity and stability

**Figure 4.**
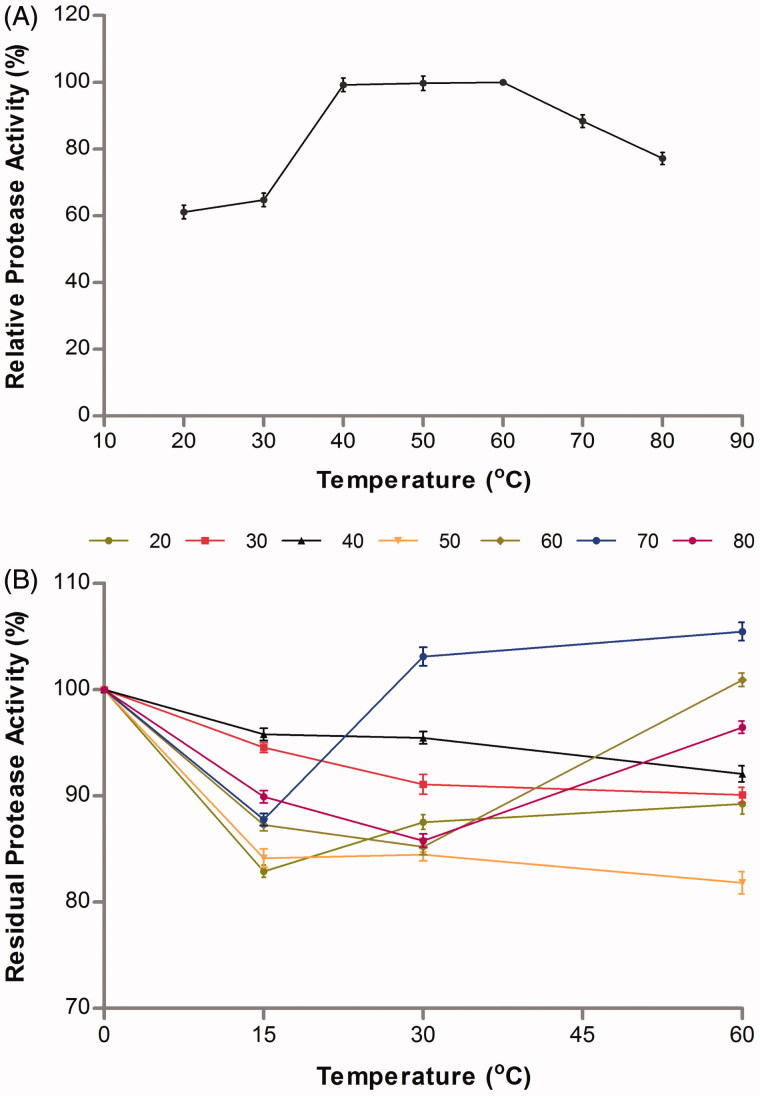
Effect of temperature on activity (A) and stability (B) of the purified protease from *A. pallidus* C10.

The effect of temperature on the protease enzyme purified from *A. pallidus* C10 was examined at different temperatures, and the optimum temperature was determined to be 60 °C. It was ascertained that C10 protease enzyme was active at a broad temperature range, from 40 to 80 °C, and that it had 99, 99, 88 and 77% activity, respectively, at the temperatures of 40, 50, 70 and 80 °C (Figure 4(A)). These results suggest that this enzyme is a thermophilic protease. Some proteases, the optimum temperature of which was 60 °C, have also been reported, which are *B. licheniformis* ATCC 21424^47^, *B. mojavensis* A21^8^, *Bacillus* sp. SM2014^46^, and *A.veronii* PG01^48^. Nevertheless, the alkaline proteases with lower optimum temperature value than the purified protease were reported in the previously-conducted studies, as well^27^^,^ 39 ^,^ 44.

The thermal stability of the protease enzyme purified from *A. pallidus* C10 was determined by performing an activity measurement at 20–80 °C temperature range at 15, 30, and 60 min. When the temperature stability profile was examined, it was determined that the enzyme had not lost more than 80% of its activity at all temperatures (Figure 4(B)). Most of the cold-active alkaline proteases fail to meet industrial requirements due to the fact that they are not stable at the temperatures above 20 °C and due to their low-product yields in large-scale productions^49^^,^ 50. On the other hand, most of the thermostable proteases lose their stability at low temperatures in spite of the fact that they show catalytic efficiency and stability at higher temperatures^8^^,^ 51. Therefore, the alkaline proteases stable at a quite broad temperature range, which are capable of remaining stable under hot, normal and cold conditions, have quite an important place in terms of industry. It is seen that *A. pallidus* C10 protease purified in this study is a powerful candidate for this sector as it is capable of preserving more than 80% of its activity at a broad temperature range, from 20 to 80 °C. Gupta and Khare^52^, in a study they conducted on *P. aeruginosa* PseA, stated that the activity that remained from the initial activity at the end of 30 minutes and at 45, 50, 55 °C proved to be 100%, 91%, and 80%. Despite this, the protease enzyme purified from *A. pallidus* C10 had preserved 92% of its initial activity at the end of 1 h at and at 40 °C, while it preserved 81% of its initial activity at the end of 1 h and at 50 °C. On the other hand, Charles et al.^39^ determined that the protease enzyme obtained from *A. nidulans* HA-10 had showed the lowest activity at 70 °C as the result of 1 h-incubation and that it had, later, become inactive at 80 °C. Heidari et al.^53^, with their study, reported that the enzyme they had purified had lost its activity when the temperature was above 60 °C. Divakar et al.^48^, however, reported that the enzyme they had isolated from *A. veronii* PG01 had preserved 53% of its activity at 70 °C at the end of 1 h. Jayakumar et al.^37^, in their study, reported that the enzymes had preserved their activities by 50% at 30–70 °C temperature range at the end of 30 minutes. On the other hand, it was determined that the protease enzyme purified from *A. pallidus* C10 had showed 105% activity at 70 °C and 96% activity at 80 °C at the end of one hour. The results obtained in this study suggest that the protease enzyme purified from *A*. *pallidus* C10 exhibited a high level of stability against temperature for quite a long time, and when compared with the other studies found in the literature, its tolerance to temperature is seen to be quite high.

### Kinetic parameters of alkaline protease

By using different concentrations of casein, *K*_M_ and *V*_max_ values for the alkaline protease purified from *A. pallidus* C10 were determined as 0.197 mg/mL and 7.29 μmol.mL^−^^1^.min^−^^1^, respectively. When compared with the alkaline proteases reported in the literature, such as *B. lehensis* (*K*_M_ of 0.35 mg.mL^−^^1^)^38^, *B. circulans* M34 (K_M_ of 0.96 mg.mL^−^^1^)^54^, *P. aeruginosa* PseA (*K*_M_ of 2.69 mg.mL^−^^1^)^52^, *B. circulans* (K_M_ of 0.597 mg.mL^−^^1^)^26^, and haloalkalophilic *Bacillus* sp. (*K*_M_ of 2 mg.mL^−^^1^)^55^, *A. pallidus* C10 alkaline protease was determined to have a higher affinity for the casein substrate. When the *V*_max_/*K*_M_ value is quite high, it means that this enzyme has a powerful catalytic activity to hydrolyze the substrate^54^. The fact that *A. pallidus* C10 protease has also a high affinity and catalytic efficiency is an important characteristic for various biotechnological applications.

### Substrate specificity of alkaline protease

As the result of the activity measurements performed by using 0.65% (w/v) BSA, casein, azocasein, gelatin and hemoglobin for the purpose of determining the specificity of the alkaline protease enzyme purified from *A. pallidus C10* to the natural substrates, the enzyme was determined to have shown the highest activity in the presence of casein (100%). The specificity of the enzyme in the face of the other substrates was determined as: gelatin (25%), azocasein (15%), BSA (5%) and hemoglobin (0%). Similarly, the alkaline proteases like *B. circulans* MTCC 7942^32^, *B. pumilus* MCAS8^37^, *A. veronii* PG01^48^, and *B. laterosporus-*AK1^41^ were also reported to have shown the highest activity against casein.

### The effect of inhibitors and metal ions on alkaline protease activity

The effect of different inhibitors on the protease enzyme obtained from *A. pallidus* C10 has been shown in Table 2. The fact that it is totally inhibited in the presence of 5 mM PMSF suggests that the purified enzyme belongs to serine protease family. In the presence of DTNB, a weak inhibition was determined. Separately, the increase in the enzyme activity in the presence of β-mercaptoethanol suggests that the enzyme is thiol dependent^56^. The fact that the protease enzyme shows high stability in the presence of EDTA is of great significance in terms of being used in the detergent industry, since the chelating agents like EDTA are among the most commonly found components in detergent formulations, which are used for the purpose of softening water and assisting in removing stains^8^^,^ 32. The stability of the purified C10 protease enzyme against EDTA indicates that it has a great advantage in terms of being the desired situation for detergent formulation and in terms of the use of the enzyme in the detergent industry due this characteristic.

**Table 2.** The effect of inhibitors on *A. pallidus* C10 alkaline protease activity.

Inhibitor	Concentration(mM)	Residual activity (%)	*p* Values
Control	0	100	–
PMSF	1	69.54 ± 0.29	<0.0001^a^
	5	0	–
EDTA	1	79.92 ± 0.08	<0.0001^a^
	5	100.4 ± 0.42	0.1277^ns^
DTNB	1	92.43 ± 0.26	<0.0001^a^
	5	77.21 ± 0.05	<0.0001^a^
β-mercaptoethanol	1	119 ± 0.57	<0.0001^a^
	5	605.7 ± 0.33	<0.0001^a^

*p* > 0.05 (not significant, ns).

a *p* < 0.0001.

When the effect of the metal ions on the protease enzyme purified from *A. pallidus* C10 was examined; it was seen that the enzyme had enhanced its activity in the presence of 1, 5, 10 mM of Ca^2+ ^ve Mg^2+ ^metal ions, whereas in the presence of Co^2+ ^and Mn^2+^, almost more than 80% of the enzyme activity was seen to have remained. However, at 10 mM concentrations in which 1 and 5 mM Ag^+ ^and K^+ ^metal ions had activated the enzyme, the activity of the enzyme was determined to have decreased by 10%. 1 mM Fe^2+ ^and Ni^2+ ^ions of the enzyme were determined to have been activated by 5 and 10 mM Zn^2+ ^(Table 3). Similarly, it was reported in the literature that the protease enzyme had been activated in the presence of Ca^2+ ^and Mg^2+ ^ions^8^^,^ 26 ^,^ 29 ^,^ 32 ^,^ 48.

**Table 3.** The effect of metal ions on *A. pallidus* C10 alkaline protease activity.

Metal ions	Concentration(mM)	Residual activity (%)	*p* Values
Control	0	100	–
Fe^2+^	1	109 ± 2.50	0.0032^b^
	5	95.41 ± 5.68	0.2692^ns^
	10	101.2 ± 1.50	0.1647^ns^
Mg^2+^	1	120.8 ± 0.38	<0.0001^d^
	5	113.1 ± 1.83	0.0003^c^
	10	113.6 ± 0.52	<0.0001^d^
Ca^2+^	1	115.3 ± 2.17	0.0003^c^
	5	120.6 ± 0.24	<0.0001^d^
	10	108.8 ± 0.69	<0.0001^d^
Zn^2+^	1	83.11 ± 7.28	0.0172^a^
	5	114.8 ± 1.45	<0.0001^d^
	10	111.6 ± 2.67	0.0016^b^
Co^2+^	1	96.61 ± 0.97	0.0093^b^
	5	87.45 ± 1.67	0.0003^c^
	10	83.08 ± 7.77	0.0212^a^
Ni^2+^	1	107.1 ± 0.77	0.0002^c^
	5	94.30 ± 1.79	0.0079^b^
	10	91.54 ± 1.64	0.0013^b^
Mn^2+^	1	83.08 ± 7.77	0.0212^a^
	5	84.92 ± 0.69	<0.0001^d^
	10	80.58 ± 4.39	0.0017^b^
Ag^+^	1	108.2 ± 0.57	<0.0001^d^
	5	124.3 ± 2.70	0.0001^c^
	10	90.78 ± 8.61	0.1501^ns^
K^+^	1	149.3 ± 5.23	<0.0001^d^
	5	129.9 ± 4.61	0.0004^c^
	10	91.60 ± 2.29	0.0041^b^

*p* > 0.05 (not significant, ns).

a *p* < 0.05.

b *p* < 0.01.

c *p* < 0.001.

d *p* < 0.0001.

### The effect of some organic solvents on alkaline protease stability

The effects of various organic solvents at different concentrations (15%, 25% and 50%, v/v) on the purified *A. pallidus* C10 protease stability are summarized in Table 4. In the wake of 1 h-incubation; it was determined that the enzyme had preserved more than 85% of its activity at 15% concentration in ethanol, acetone, DMSO, butanol, chloroform and isopropanol solutions, whereas ethanol and methanol solutions with 25% concentration had enhanced the enzyme activity, and in other organic solutions; however, it did not lose its activity at the rate of almost 70–80%. Nonetheless, it was ascertained that the enzyme retained more than 80% of its activity at 50% concentration in the presence of DMSO and methanol, and at the rate of almost 50% in butanol, chloroform and isopropanol solutions. After 24 h; it was seen that the enzyme had lost its activity at the rate of 10–26% at 15% concentrations in the presence of methanol, ethanol, isopropanol and chloroform, and again, it lost its activity at the rate of 4–50% at 25% concentrations. Enzyme activity was found to be induced by acetone (15%), DMSO (25%), and butanol (25%) by 103%, 102% and 106%, respectively. At 50% concentrations; however, the enzyme was determined to have failed to preserve its stability in the organic solvents mentioned. The organic solvent stability of *A. pallidus* C10 protease is higher than the other alkaline proteases mentioned, which are *Bacillus* sp. NPST-AK15^1^, *Saccharopolyspora* sp. A9^57^, and *B. pumilus* MCAS8^37^. In the presence of organic solvents, the ability of the natural proteases to remain stable without performing any modification for the stabilization of the enzyme is of great importance for various applications^58^. The use of proteases for peptide and ester synthesis non-aqueous conditions in the organic media has gained a great deal of importance within the last decade^59^. Since it exhibits good stability in the presence of organic solvents, the protease enzyme obtained from *A. pallidus* C10 can be said to have a great advantage in terms of its applicability in these industrial applications.

**Table 4.** The effect of organic solvents on *A. pallidus* C10 alkaline protease activity.

Organic solvents	Concentration (%)	Residual activity (%) (1h)	*p* Values	Residual activity (%) (24h)	*p* Values
Control	0	100	–	100	–
Methanol	15	68.42 ± 1.83	<0.0001^d^	83.38 ± 1.77	0.0001^c^
	25	114 ± 2.79	0.0009^c^	50.27 ± 1.39	<0.0001^d^
	50	80.24 ± 1.74	<0.0001^d^	0	–
Ethanol	15	93.59 ± 1.12	0.0011^b^	90.28 ± 1.98	0.0014^b^
	25	107.8 ± 1.52	0.0009^c^	75.47 ± 2.95	0.0002^c^
	50	103.7 ± 2.07	0.0303^a^	0	–
Acetone	15	86.73 ± 1.29	<0.0001^d^	102.9 ± 1.92	0.0472^a^
	25	88.50 ± 2.02	0.0008^c^	72.67 ± 1.45	<0.0001^d^
	50	72.74 ± 1.89	<0.0001^d^	0	–
DMSO	15	84.73 ± 0.93	<0.0001^d^	85.86 ± 3.65	0.0030^b^
	25	78.10 ± 1.75	<0.0001^d^	102.2 ± 1.53	0.0522^ns^
	50	95.33 ± 2.19	0.0299^a^	0	–
Butanol	15	93.74 ± 2.09	0.0091^b^	58.88 ± 2.20	<0.0001^d^
	25	73.66 ± 1.85	<0.0001^d^	106.5 ± 3.21	0.0217^a^
	50	52.01 ± 2.44	<0.0001^d^	0	–
Chloroform	15	95.94 ± 2.58	0.0724^ns^	74.56 ± 0.13	<0.0001^d^
	25	85.02 ± 1.73	0.0002^c^	96.41 ± 0.87	0.0056^b^
	50	59.59 ± 1.44	<0.0001^d^	0	–
Isopropanol	15	90.21 ± 1.91	0.0012^b^	90.13 ± 1.73	0.0008^c^
	25	84.46 ± 1.00	<0.0001^d^	62.91 ± 2.42	<0.0001^d^
	50	50.50 ± 1.038	<0.0001^d^	0	–

*p* > 0.05 (not significant, ns).

a *p* < 0.05.

b *p* < 0.01.

c *p* < 0.001.

d *p* < 0.0001.

### The effects of surfactants, oxidizing agent and commercial detergents on alkaline protease stability

It was determined that the protease enzyme purified from *A. pallidus* C10 could not preserve its stability in the presence of all the compounds studied on apart from SDS. While the enzyme showed weak stability in the presence of the oxidizing agent, H_2_O_2_, it was determined to have preserved its activity by 79% at 1% SDS concentration, and it enhanced its activity by 16% in the presence of 5% SDS (Table 5). While the number of studies in the literature in which stable enzymes are found in the presence of SDS are few^37^^,^ 38 ^,^ 46, the fact that the protease enzyme purified in this study has high stability in the presence of SDS is important.

**Table 5.** The effect of various surfactants and the oxidant agent on *A. pallidus* C10 alkaline protease activity.

Surfactants/oxidizingagent	Concentration(%) (v/v)	Residual activity (%)	*p* Values
Control	0	100	–
SDS	1 (w/v)	79.33 ± 2.65	0.0002^a^
SDS	5 (w/v)	116.7 ± 1.76	<0.0001^b^
Triton X-100	1	41.33 ± 1.85	<0.0001^b^
Triton X-100	5	63.0 ± 1.52	<0.0001^b^
Tween-20	1	20.0 ± 1.15	<0.0001^b^
Tween-20	5	37.67 ± 1.45	<0.0001^b^
Tween-80	1	31.0 ± 2.08	<0.0001^b^
Tween-80	5	29.0 ± 2.08	<0.0001^b^
H_2_O_2_	1	9.66 ± 0.88	<0.0001^b^
H_2_O_2_	5	11.67 ± 0.88	<0.0001^b^

a *p* < 0.001.

b *p* < 0.0001.

**Figure 5.**
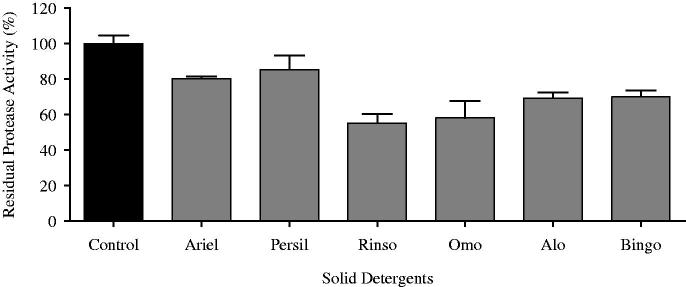
Stability of the alkaline protease from *A. pallidus* C10 in the presence of various commercial detergents.

As seen in Figure 5, it was ascertained that the enzyme had not lost its activity by 80.25% and 85.28%, respectively, in the presence of Ariel and Persil; whereas, in the presence of other detergents, it was seen to have preserved more than 55% of its initial activity. Rai and Mukherjee^27^ reported that *B. subtilis* DM-04 protease enzyme had showed 60% activity in the presence of Ariel at 37 °C during 1 h incubation. Joshi and Satyanarayana^38^ also determined that *B. lehensis* protease enzyme had preserved its activity by 80% in 0.5% Ariel solution and in 1% Ariel solution.

## Conclusion

Alkaline proteases are among the most important hydrolytic enzymes, which have commercial value and which have several fields of practice in various industrial sectors. In this study, the alkaline protease enzyme produced by a new strain called *A. pallidus* C10 was purified and characterized by using two different chromatographic methods. Although there are studies found in the literature with respect to *A. pallidus* species and the identification of the isolates belonging to this species, our study is the first one in which the alkaline protease enzyme was purified and characterized. It was determined that the enzyme had become highly active and stable under alkaline conditions (pH 7.0–10.0) and that it had preserved its activity to a large extent at a broad temperature range (20–80 °C). It was ascertained to be more stable particularly at high temperatures like 60 °C, 70 °C and 80 °C when compared with similar studies conducted before. Thus, it is seen that the purified protease enzyme, with this aspect, can be the candidate for reason of preference for the commercial applications that require long-term stability at high temperatures. Separately, the enzyme was observed to have enhanced or preserved its activity in the presence of various metal ions, EDTA, SDS and organic solvents. When all these data are considered together, they indicate to the applicability potential of the thermostable *A. pallidus* C10 alkaline protease as a great detergent additive in various industrial fields and biotechnological applications, notably the detergent industry.
